# Implications of Palliative Care Screening in Outpatient Comprehensive Cancer Genome Profiling Tests

**DOI:** 10.31662/jmaj.2025-0137

**Published:** 2025-09-05

**Authors:** Banri Tsuda, Yuko Ohnuki, Hiromi Tomomatsu, Miho Ito, Yuki Takahashi, Mizuho Suzuki, Ai Unzaki, Tomoari Mori, Makoto Tokuhara, Kei Takeshita

**Affiliations:** 1 Department of Palliative Care Medicine, Tokai University School of Medicine, Tokai University School of Medicine, Isehara, Kanagawa Prefecture, Japan; 2 Department of Medical Ethics, Tokai University School of Medicine, Isehara, Kanagawa Prefecture, Japan; 3 Department of Clinical Genetics, Tokai University Hospital, Isehara, Kanagawa Prefecture, Japan

**Keywords:** comprehensive cancer genome profiling tests, palliative care, medical ethics, palliative medicine, clinical genetics

## Abstract

**Introduction:**

Many patients with cancer experience physical and/or psychological distress when undergoing Comprehensive Cancer Genome Profiling (CGP) after being diagnosed with cancer. However, not all of these patients necessarily visit palliative care clinics. This study aimed to clarify the extent to which patients who underwent the CGP test experience physical and emotional distress.

**Methods:**

The “Ease of Living Questionnaire” was administered to 75 patients who visited our CGP outpatient clinic between November 2022 and July 2023, and patient backgrounds were investigated.

**Results:**

Of the 75 patients, 8 (16%) had physical symptoms graded as ≥3/5, and five had visited a palliative care outpatient clinic. On the other hand, 14 patients (28%) had psychological symptoms graded as ≥6/10, but only eight visited the palliative care outpatient clinic.

**Conclusions:**

Several patients who underwent CGP examinations at our hospital experienced physical and psychological distress, suggesting a strong need for palliative care. However, the number of patients who undergo interventions by palliative care physicians is not necessarily high. It is important to take advantage of the fact that CGP outpatient clinics provide sufficient time for healthcare professionals to meet with patients and their families, as well as to seek to connect patients in need of palliative care with appropriate palliative care CGP outpatient clinics in the future.

## Introduction

Comprehensive Cancer Genome Profiling (CGP) is widely used to improve the prognosis of patients with advanced cancer. In Japan, the Cancer Gene Panel Test was approved in December 2018, insurance coverage was obtained in June 2019, and the provision of genomic medicine began ^(1)^. The following conditions must be satisfied to perform the CGP test: (1) the patient is expected to complete standard treatment or has solid tumors for which standard treatment standard therapy is not available; (2) the patient has a performance status that allows treatment to be conducted if recommended by a molecular tumor committee (expert panel); (3) the CGP tests are conducted at designated hospitals or cooperating hospitals designated by the Ministry of Health, Labour and Welfare that are responsible for training and research in cancer genome medicine and holding expert panels ^(2)^; (4) prior informed consent is obtained; (5) all test results are discussed by the expert panel; and (6) patient consent, if obtained, includes permission to submit clinical and genomic data to the Center for Cancer Genome Information and Advanced Therapeutics ^(3)^.

In this study, the setting was a hospital that is designated as a regional hospital for cancer treatment. Therefore, it is mandated to screen patients with cancer for physical, psychological, and social distress from the time of diagnosis in both outpatient clinics and hospital wards ^(4)^. The Ease of Living Questionnaire is one of the initial screening methods for patients with cancer in need of pain relief ^(5)^. It is a patient-administered questionnaire that assesses the quality of life and needs of patients undergoing outpatient chemotherapy, and there are multiple reports on its validity ^(6), (7), (8)^. If the patient’s physical or psychological distress is likely to exceed a certain level, a visit to a palliative care outpatient clinic is suggested. However, the Ease of Living Questionnaire is completed by patients in the early stages of treatment, and no system has been established to assess pain and psychological distress caused by cancer as anticancer drug treatment progresses. In particular, CGP testing is covered by health insurance for patients who have completed standard treatment or are expected to complete it; physical and psychological distress in these patients may differ significantly from that at the start of treatment.

This study aimed to clarify the extent to which patients who underwent the CGP test experience physical and emotional distress using the Ease of Living Questionnaire in a genetics outpatient clinic, where the CGP test was explained.

## Materials and Methods

**Figure 1.**
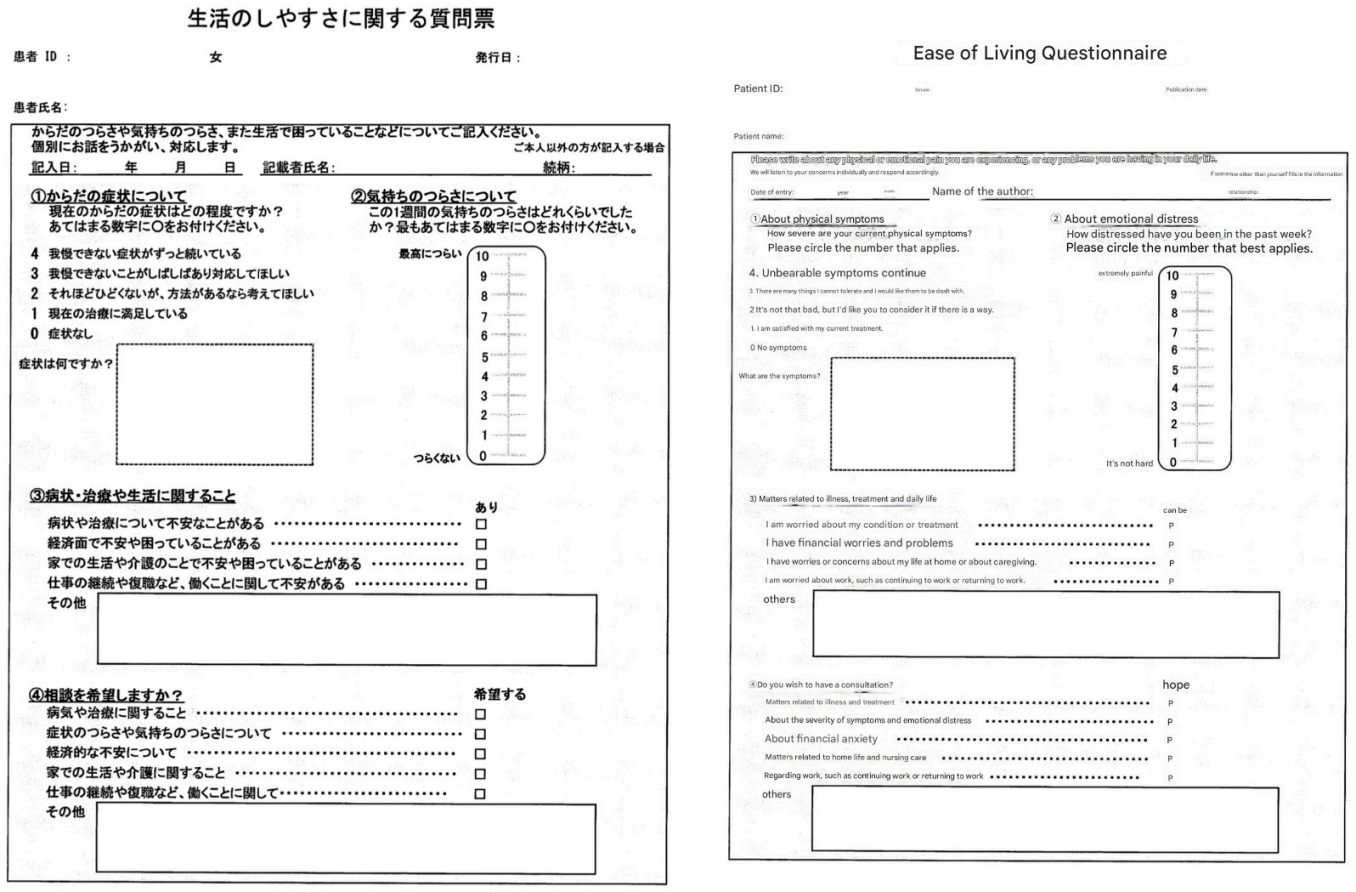
Ease of Living Questionnaire at Tokai University Hospital (in Japanese) The questionnaire is as follows: Please describe your physical and emotional pain, as well as any difficulties you are experiencing in your life. (1) About your physical symptoms What are your current physical symptoms? Please circle the number that applies. 4 Symptoms that I cannot endure have been continuing for a long time. 3 I often have problems that I cannot endure, and I want you to take care of them. 2 It is not that bad, but if there is a way to improve the symptoms, please let me know. 1 I am satisfied with my current treatment. 0 No symptoms What are your symptoms? (Free answer) (2) About emotional distress How distressed have you been during the past week? Please circle the number that applies most to you. 10 Most painful 0 Not painful (3) Symptoms, treatment, and life-related issues (check boxes) I have concerns about symptoms and treatment. I have concerns or problems with finances. I have worries or problems about living at home or caring for my family. I have concerns about working, such as continuing to work or returning to work. Others (free answers) (4) Do you want to have a consultation? (check box) Matters related to illness or treatment About the physical or emotional distress Financial concerns Living at home or nursing care About continuing to work, returning to work, etc. Others (free answers).

This study included patients who visited the CGP outpatient clinic at our hospital from November 2022 to June 2023 and completed the Ease of Living Questionnaire. The questionnaire is presented in Figure 1.

The patients were scheduled for a visit at the CGP outpatient clinic by the attending physician. Patients referred from other hospitals secured their appointments through the Medical Cooperation Office. During the patients’ visits, physicians and genome coordinators from the Department of Clinical Genetics explained the test and provided counseling. Patients completed the Ease of Living Questionnaire at the CGP outpatient clinic, followed by an outpatient visit. Based on the severity of physical symptoms and feelings reported in the questionnaire, patients were referred to an outpatient palliative care clinic.

This study was approved by the Clinical Research Review Committee of the Tokai University School of Medicine (23R092). The study conformed with the principles outlined in Ethical Guidelines for Medical and Health Research Involving Human Subjects ^(9)^. Written informed consent for publication was obtained from all patients who participated in this study.

## Results

### Patients

Of the 75 patients who visited the CGP outpatient clinic at our hospital between November 2022 and July 2023, 60 patients (34 men [52%] and 31 women [48%]; mean age, 66 years; age range, 41-88 years) who could be traced were included in the study.

### Departments referring patients to the CGP outpatient clinic

**Figure 2.**
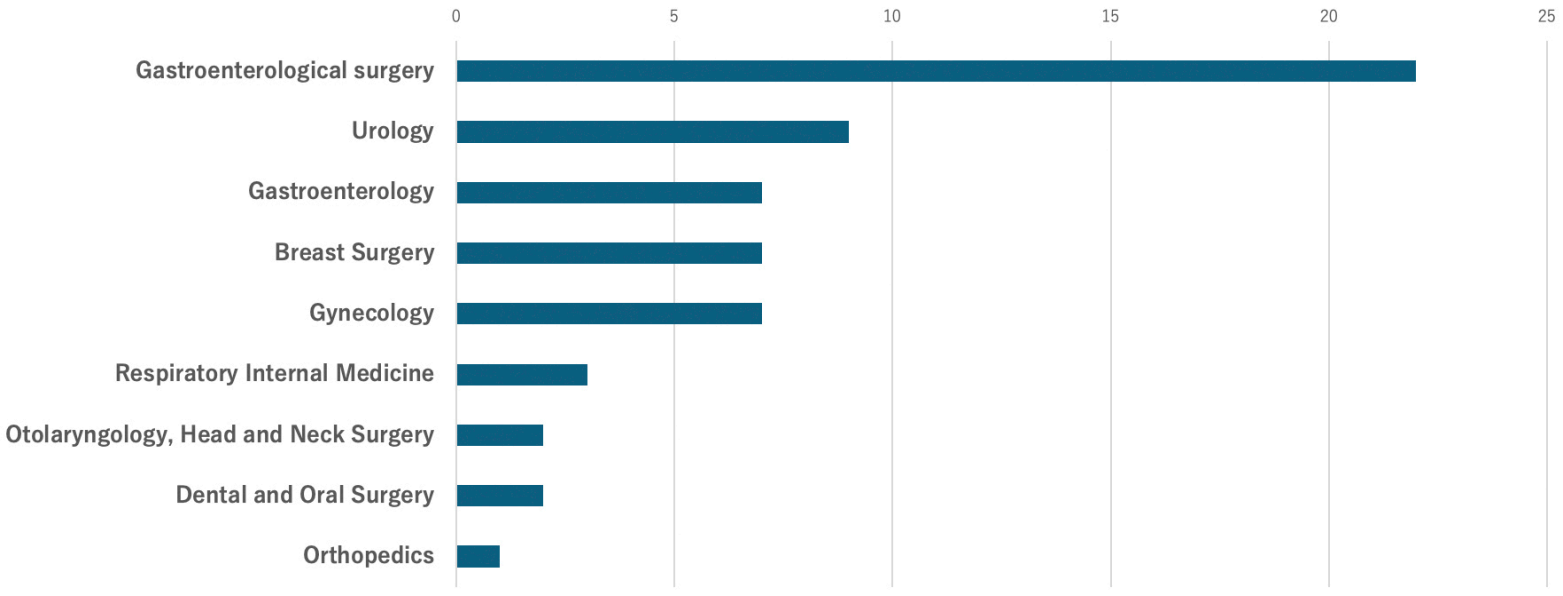
Departments referring patients to the CGP outpatient clinic. Gastroenterological surgery was the most common referral source within the hospital (22 cases, 36%), followed by urology (9 cases, 15%). CGP: Cancer Genome Profiling.

The largest number of referrals from the hospital came from gastroenterological surgery, with 22 cases (36%). Urology was the second most common referral with nine cases (15%), followed by gastroenterology, breast surgery, and gynecology. Fifteen patients were referred from other hospitals (Figure 2).

### Reasons for visiting the CGP outpatient clinic

**Figure 3.**
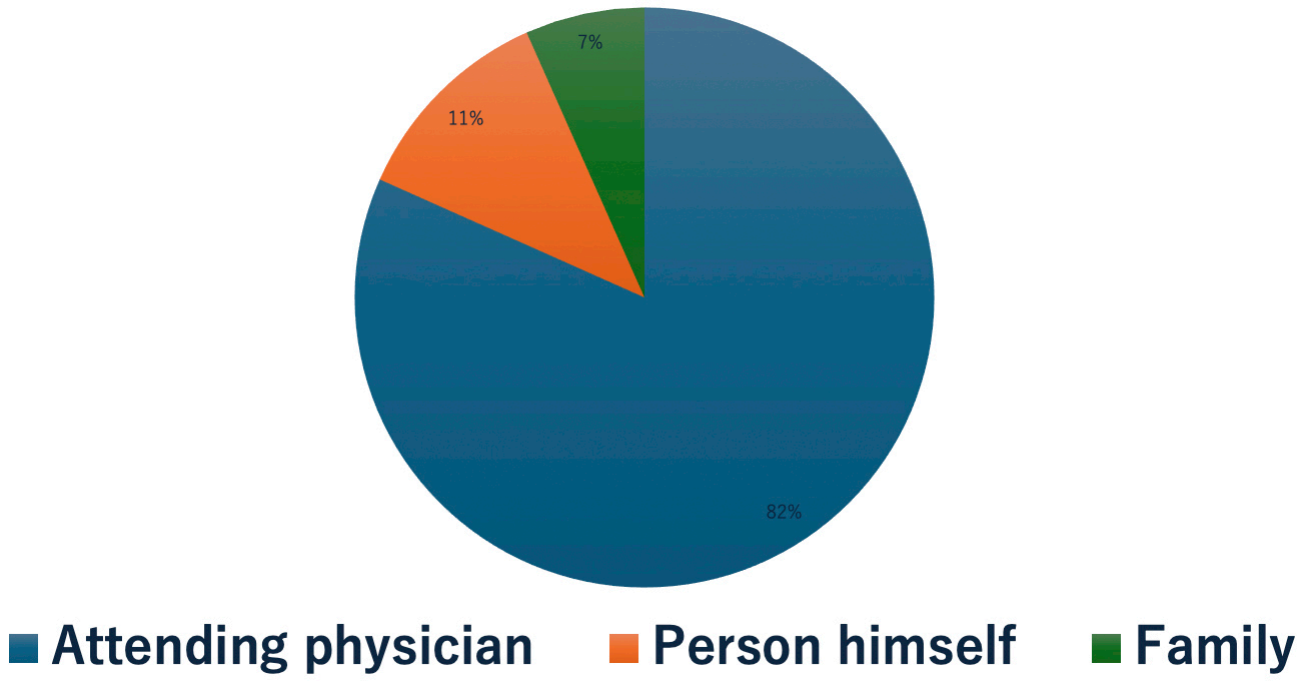
Reasons for visiting to CGP outpatient clinic. The primary physician made the recommendations in 49 cases (82%). Patients made the request in seven cases (11%), and family provided the recommendation in four cases (7%). CGP: Cancer Genome Profiling.

The most common reason for visiting a CGP outpatient clinic was a recommendation from the attending physician in 49 cases (82%), with seven cases (11%) requested by the patients themselves and five cases (7%) by family members (Figure 3).

### Physical distress

**Figure 4.**
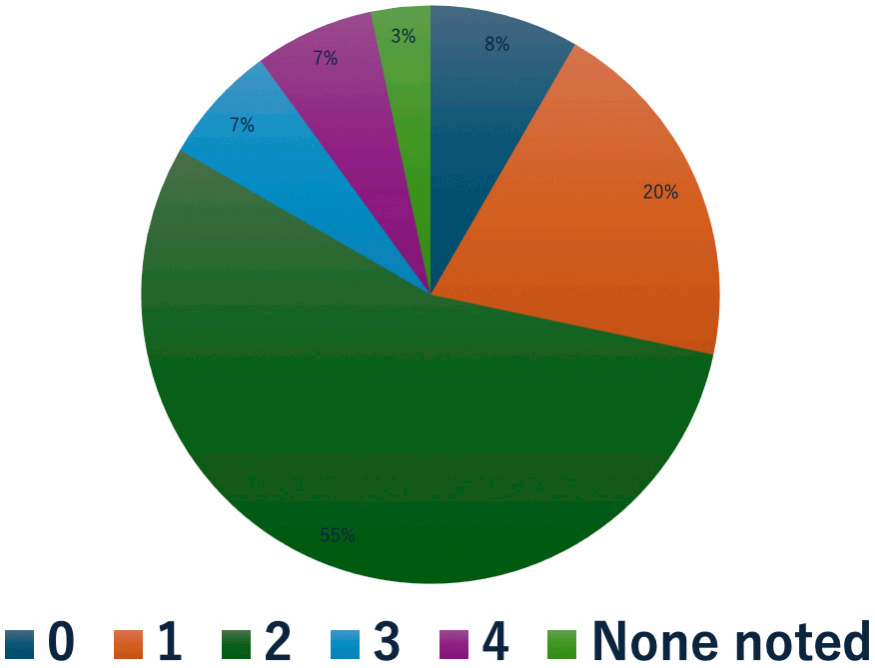
Pie chart showing physical distress (0, no symptoms; 4, unbearable distress persists). Eight patients (13%) had physical symptoms graded as ≥3 on a 5-point scale. Four patients had visited the palliative care outpatient clinic before visiting the CGP outpatient clinic, one patient was referred from the CGP outpatient clinic, one patient did not visit the CGP outpatient clinic, and two patients visited outside the study period. CGP: Cancer Genome Profiling.

The severity of physical symptoms was rated on a 5-point scale from 0 to 4. Eight patients (13%) had a severity score of ≥3 out of 5 (Figure 4). Of the eight patients, four had been seen in the palliative care outpatient clinic prior to their CGP outpatient visit, one was referred from the CGP outpatient clinic, one was recommended to see the palliative care outpatient clinic but did not, and two were seen outside the study period.

### Emotional distress

**Figure 5.**
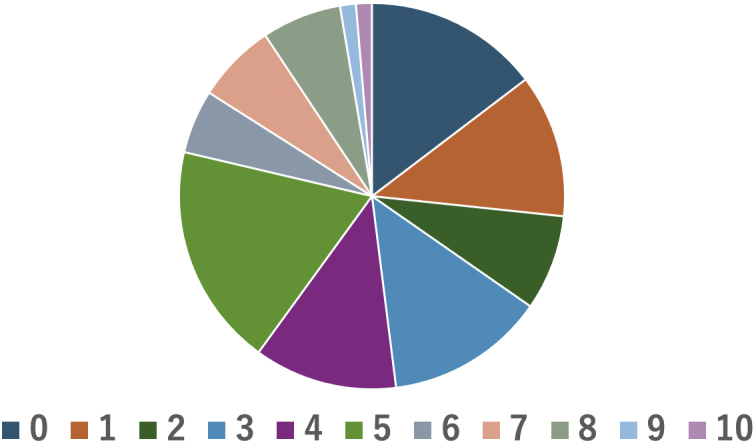
Pie chart showing emotional distress (0, light; 10, heavy). Sixteen patients (27%) had a score ≥6 on a 10-point scale. Five patients visited the palliative care outpatient clinic before they visited the CGP outpatient clinic. Three patients were referred from the CGP outpatient clinic, four were not seen, and one visited outside the study period. CGP: Cancer Genome Profiling.

In 16 cases (27%), the level of feeling of vexation was ≥6 on a scale of 10 (Figure 5). Five patients had been seen in the palliative care outpatient clinic prior to their CGP outpatient visits, three patients were referred from CGP outpatient visits to the palliative care outpatient clinic, four patients were referred but not seen, and one patient was seen outside the study period.

### Patients referred to the palliative care outpatient clinic from the CGP outpatient clinic

Table 1 shows details of patients with high levels of physical symptoms and emotional distress, showing those referred to the palliative care outpatient clinic from the CGP outpatient clinic and those who did not attend.

**Table 1.**
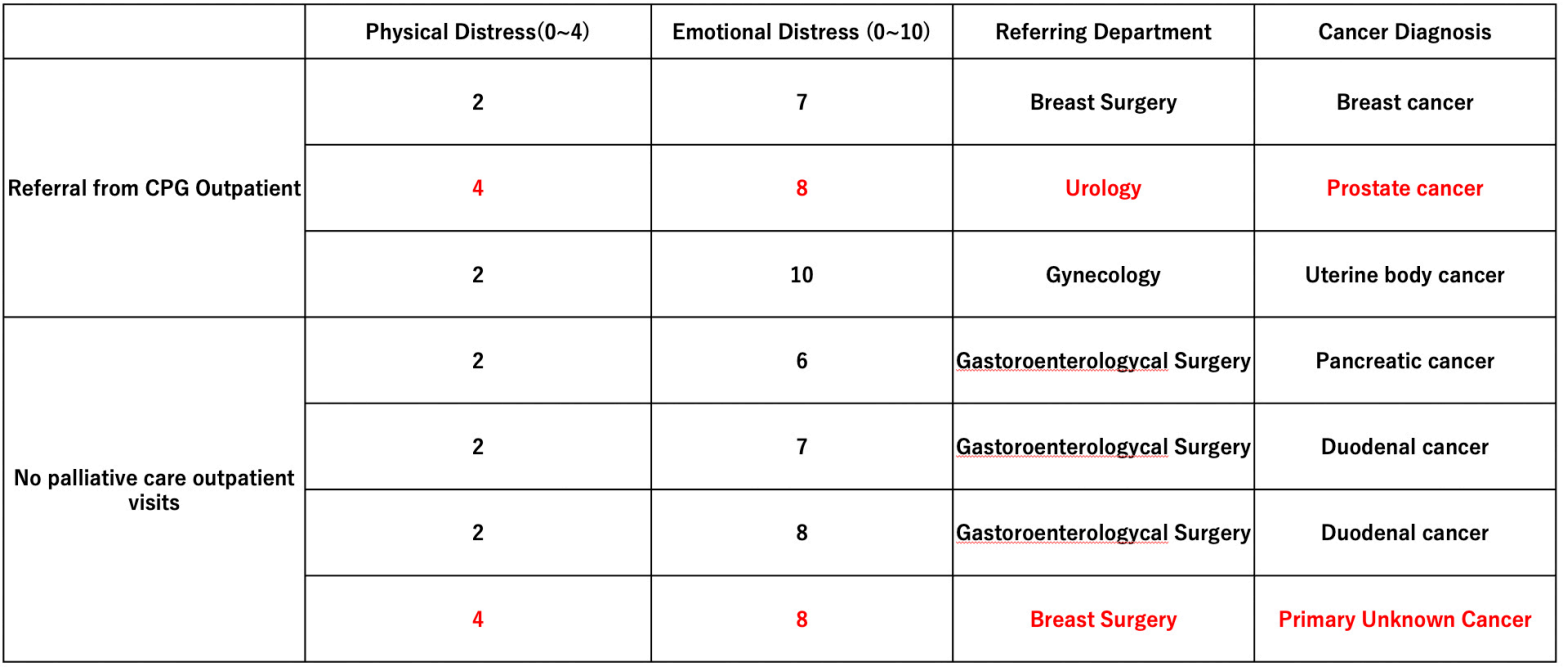
Details of patients who were referred to the palliative care outpatient clinic from the CGP outpatient clinic and those who were not seen. It should be noted that two patients had both physical and emotional distress, but one visited the palliative care outpatient clinic at the suggestion of the CGP outpatient clinic, whereas the other did not (shown in red). CGP: Cancer Genome Profiling.

Three patients were referred to the palliative care outpatient clinic from the CGP outpatient clinic. Of these, two patients showed less severe physical pain but more severe emotional distress. However, one patient with prostate cancer, referred from urology, showed high levels of both physical and emotional distress (shown in red). The patient was a man in his 70s with locally advanced prostate cancer treated with maximal androgen deprivation therapy. Estramustine sodium phosphate hydrate was then used, and finally, docetaxel was administered.

Four patients were evaluated at the CGP outpatient clinic for severe distress, either physical or emotional, and were advised to visit the palliative care outpatient clinic, but did not. Three patients did not exhibit severe physical symptoms but had severe emotional distress. One patient with a primary unknown cancer, referred by a breast surgeon, exhibited both severe physical and emotional distress (shown in red). This patient was a woman in her 60s with cancer of unknown primary origin (peritoneal carcinoma) and concurrent cancerous pleurisy and peritonitis. She was treated with six cycles of docetaxel + carboplatin chemotherapy followed by maintenance treatment with niraparib.

## Discussion

To the best of our knowledge, this is the first study in which findings from a questionnaire on the ease of living were revisited during a CGP test. The results showed that there were still patients who had been treated with standard care who did not visit the palliative care outpatient clinic during treatment, although they had physical or psychological distress that warranted a visit. At the time of CGP outpatient visits, patients were more likely to suffer from emotional distress than physical symptoms, suggesting that the patients were more burdened by emotional distress about the end of standard treatment and the low probability of receiving further successful treatment than by physical symptoms caused by chemotherapy side effects or by the primary disease. The burden on patients was considered to be due to the emotional stress of seeking treatment with a low probability of success, including clinical trials.

Approximately half of the patients had already been referred to or visited a palliative care outpatient clinic at the time of their CGP outpatient visit. Many of these patients were referred to a palliative care outpatient clinic by their attending physicians when they visited the CGP outpatient clinic. However, some patients were referred to the palliative care outpatient clinic for the first time at the time of the CGP outpatient visit because of emotional distress or physical symptoms, and some patients did not visit the palliative care outpatient clinic even though it was suggested at the CGP outpatient clinic. The reason for this may be based on medical factors. However, as symptoms change throughout the disease, there is a need for repeated screening rather than only at the time of the visit. Additionally, although the number of cases was small, the possibility that the intentions of the department or attending physician may have influenced the results cannot be ruled out.

Personal factors may also explain patients’ not visiting the palliative care outpatient clinic, despite being suggested to do so at the CGP outpatient clinic. Possible reasons include: palliative care interferes with cancer treatment; cancer treatment takes precedence over pain treatment; unwillingness to admit that the cancer is progressing; unwillingness to admit that death is near; when visiting the palliative care outpatient clinic, patients are advised to use medical narcotics but do not want to use them; not wanting to cause concern to others by visiting the outpatient palliative care clinic; not wanting to tell their doctor that they have been referred to the palliative care department; and not thinking that seeing a palliative care department will help with pain control ^(10), (11), (12), (13), (14), (15)^.

Some limitations of this study should be noted. First, only a small sample of cases from one institution was included; the results may change with a larger sample size and number of institutions. Second, this study defined significant distress as 3/5 or greater for physical symptoms, and 6/10 or greater for emotional symptoms; however, these definitions are subjective and thus debatable. Notably, preconceptions about palliative care are likely to be barriers to intervention. We should continue to accumulate cases to address these issues and build a system that can identify patients in need of palliative care intervention.

Some patients who undergo CGP examinations at our hospital have physical and emotional distress, suggesting that they have a high need for palliative care. At present, we are seeking to establish a system to provide appropriate palliative care through the CGP outpatient clinic, taking advantage of the fact that this clinic provides sufficient time for medical staff to interact with patients and their families. It is desirable to establish a system in which all patients who require palliative care interventions can be seen at the palliative care outpatient clinic before CGP outpatient visits.

## Article Information

### Acknowledgments

We thank Editage for proofreading the manuscript and providing language editing support. We are grateful to all those who contributed to this study, including colleagues for their valuable discussions and the staff at Tokai University Hospital for their support.

### Author Contributions

Banri Tsuda and Yuko Ohnuki conceptualized the study, designed the experiments, and wrote the manuscript. Yuko Ohnuki conducted the experiments and analyzed the data. All authors contributed to data interpretation and manuscript revision. All authors reviewed and approved the final manuscript.

Yuko Ohnuki is acknowledged as a joint first author, having contributed equally to this work.

### Conflicts of Interest

None

### IRB Approval Code and Name of the Institution

This study was approved by the Clinical Research Review Committee of the Tokai University School of Medicine (approval number: 23R092).
